# Behavioral and Sensory Deficits Associated with Dysfunction of GABAergic System in a Novel *shank2*-Deficient Zebrafish Model

**DOI:** 10.3390/ijms24032208

**Published:** 2023-01-22

**Authors:** Yi Wang, Chunxue Liu, Jingxin Deng, Qiong Xu, Jia Lin, Huiping Li, Meixin Hu, Chunchun Hu, Qiang Li, Xiu Xu

**Affiliations:** 1 Division of Child Health Care, Children’s Hospital of Fudan University, National Children’s Medical Center, 399 Wanyuan Road, Shanghai 201102, China; 2 Center for Translational Medicine, Institute of Pediatrics, Shanghai Key Laboratory of Birth Defect, Children’s Hopstial of Fudan University, National Children’s Medical Center, 399 Wangyuan Road, Shanghai 201102, China

**Keywords:** zebrafish, ASD, *shank2*, behavior, GABAR

## Abstract

Hyper-reactivity to sensory inputs is a common and debilitating symptom of autism spectrum disorder (ASD), but the underlying neural abnormalities remain unclear. Two of three patients in our clinical cohort screen harboring de novo *SHANK2* mutations also exhibited high sensitivity to visual, auditory, and tactile stimuli, so we examined whether shank2 deficiencies contribute to sensory abnormalities and other ASD-like phenotypes by generating a stable *shank2b*-deficient zebrafish model (*shank2b^−/−^*). The adult *shank2b^−/−^* zebrafish demonstrated reduced social preference and kin preference as well as enhanced behavioral stereotypy, while larvae exhibited hyper-sensitivity to auditory noise and abnormal hyperactivity during dark-to-light transitions. This model thus recapitulated the core developmental and behavioral phenotypes of many previous genetic ASD models. Expression levels of γ-aminobutyric acid (GABA) receptor subunit mRNAs and proteins were also reduced in *shank2b^−/−^* zebrafish, and these animals exhibited greater sensitivity to drug-induced seizures. Our results suggest that GABAergic dysfunction is a major contributor to the sensory hyper-reactivity in ASD, and they underscore the need for interventions that target sensory-processing disruptions during early neural development to prevent disease progression.

## 1. Introduction

Autism spectrum disorder (ASD) is an umbrella term for neurodevelopmental syndromes characterized by deficits in social interaction and communication, stereotyped repetitive behaviors, restricted interests, and various sensory abnormalities [1,2]. Atypical sensory processing in ASD may be manifested as hypersensitivity, hyposensitivity, or unusual interest in specific sensory aspects of the environment, and such abnormalities are observed in up to 90% of autistic individuals [2,3,4,5]. Furthermore, these abnormalities influence every sensory modality (vision, smell, taste, audition, proprioception, touch, and balance).

Atypical visual processing has been observed in human infants who eventually develop autism as early as 6 months of age [6,7], considerably earlier than core autistic phenotypes such as impairments in joint attention (14–18 months) [5,8]. Moreover, previous studies have reported associations between lower joint attention in children with autism and lack of orienting to both social and nonsocial sensory stimuli [8]. Sensory symptoms in infants not only precede but also are predictive of social deficits [9], repetitive behaviors [10], and eventual diagnosis of ASD in childhood [9]. Thus, perceptual symptoms could serve as early diagnostic biomarkers for ASD, provide clues to the underlying pathobiology, and reveal novel targets for therapeutic intervention [11,12].

Many genes have been implicated in ASDs, including those that regulate synaptic transmission [13,14]. Among these is *SHANK2* [15,16], which encodes a major scaffold protein (SHANK2/ProSAP1) at glutamatergic synapses essential for the assembly and integrity of the postsynaptic density (PSD) [13,14]. Previous studies have identified eighteen patients with neurodevelopmental disorder harboring de novo variants in the human *SHANK2* gene [15,16,17,18,19,20,21,22,23,24,25], and we identified three additional patients with de novo variants of *SHANK2* in our clinical screening cohort. Moreover, two of these patients showed intellectual deficits and heightened sensitivity to sensory stimuli, such as exaggerated reflexive responses to sudden sounds and passive seeking of external visual stimuli. In accord with clinical observations, a line of *Shank2* knockout mice also demonstrated hyposensitivity to painful stimuli [26]. Accordingly, we speculated that SHANK2 loss-of-function in autism would be associated with abnormal sensory processing, especially in the auditory and visual pathways [2]. However, the exact molecular mechanisms remain to be elucidated.

These ASD-related sensory deficits and core symptomatic behaviors have been attributed to an imbalance between excitatory and inhibitory neurotransmission [27,28,29,30,31] due to deficient inhibitory γ-aminobutyric acid (GABA) signaling. Lines of evidence for GABAergic dysfunction in ASD include underexpression of GABA receptor subunits in post-mortem brain tissue of autism patients, associations between ASD and allelic variants of GABAergic genes, and deficient GABAergic transmission in a number of otherwise distinct animal models of ASD [32,33,34,35]. Moreover, a recent human study found deficient binocular rivalry, which is dependent on cortical GABAergic signaling, in autism patients [36]. There is also evidence for the involvement of GABAergic dysfunction in the tactile and auditory processing deficits of autism [11,37,38]. Collectively, these findings indicate that altered GABAergic signaling may contribute to abnormal sensory perception in autism.

The zebrafish is a powerful tool for studying neurological diseases including ASD at the genetic, developmental, and behavioral levels [39]. Here, we report the generation of a *shank2* mutant zebrafish line that provides strong support for the contribution of shank2 deficits in the sensory processing and behavioral abnormalities of ASD. Moreover, we report that these animals exhibit reduced expression of GABA receptors in the brain and enhanced sensitivity to drug-induced seizures, suggesting GABAergic signaling insufficiency.

## 2. Results

### 2.1. Generation and Morphometric Characterization of shank2-Deficient Zebrafish

**Figure 1:**
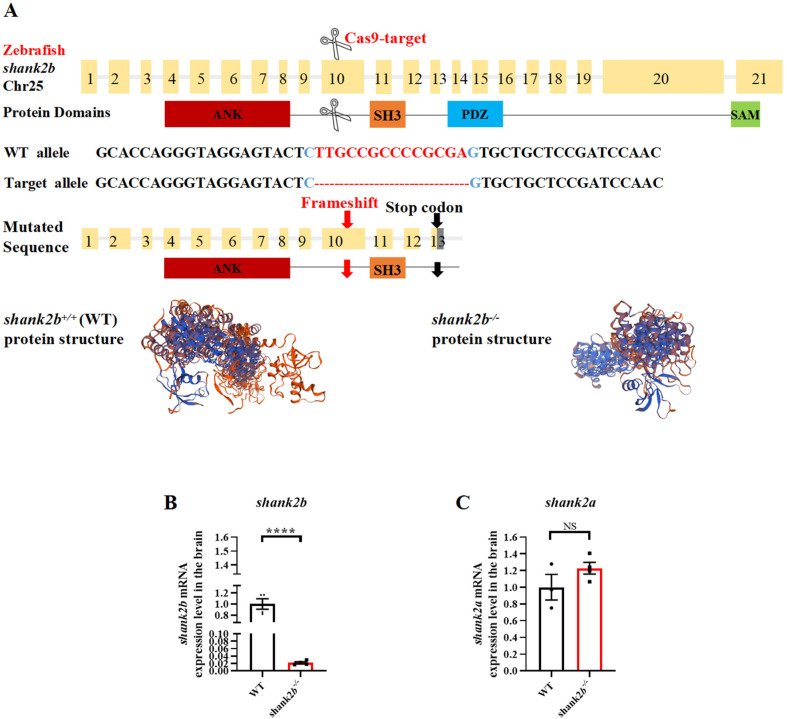
Generation of a zebrafish *shank2b* mutant using CRISPR/Cas9. (**A**) Structure of zebrafish *shank2b* gene and protein. The protein domains (ANK, ankyrin repeat domain; SH3, Src homology 3 domain; PDZ, PSD-95/Discs large/ZO-1 domain; SAM, sterile alpha motif domain) are aligned to the corresponding exons. Diagram of target site on 10th exon of zebrafish *shank2b* genomic DNA and a 14 bp deletion mutation by CRISPR/Cas9 gene editing. Predicted structures of wild-type (WT) and *shank2b* mutant proteins in zebrafish. The 14 bp deletion resulted in a stop codon on 13th exon and premature termination before the PDZ and SAM domains. (**B**) Reduced expression of *shank2b* mRNA in the brain of *shank2b^−/−^* adult male zebrafish at 4.5 months post-fertilization (mpf) compared to WT fish, analyzed by RT-qPCR (WT n = 4, *shank2b^−/−^* n = 4, **** *p* < 0.0001, Student’s *t* test). Data are shown as mean ± SEM. (**C**) The expression of *shank2a* mRNA in the brain of WT and *shank2b^−/−^* adult (4.5 mpf) male zebrafish was not affected (WT n = 3, *shank2b^−/−^* n = 4, ns, *p* = 0.1957, Student’s *t* test). Data are shown as mean ± SEM.

Zebrafish have two *shank2* paralogs that are expressed during development and continue to be enriched in the mature central nervous system (Supplementary Figure S1A,B). Shank2a and shank2b proteins show 46.63% and 60.58% identity to the human protein (Supplementary Table S2), respectively, and contain the same major functional domains (Figure 1A, Supplementary Tables S3 and S4). Moreover, a comparison of protein sequences from multiple species revealed high evolutionary conservation of SHANK2 among vertebrates (Supplementary Figure S1C).

To examine shank2 functions, we generated a *shank2b*-deficient zebrafish line using CRISPR/Cas9 to target the tenth exon of the *shank2b* gene, creating a 14-bp deletion (Figure 1A, Supplementary Figure S1D). The resulting frame shift produces a premature stop codon in exon 13 that disrupts the amino acid sequences of downstream PSD-95/Discs large/ZO-1 (PDZ) and sterile alpha motif (SAM) domains (Figure 1A).

To assess whether the mutant *shank2* transcript is subject to nonsense-mediated decay, we examined *shank2b* mRNA expression in the zebrafish brain. Quantitative RT-PCR (RT-qPCR) revealed an overall reduction in *shank2b* mRNA among homozygous mutants compared to the wild types (WT) (Figure 1B), whereas the expression of *shank2a* mRNA did not differ between genotypes (Figure 1C). The relative abundance of *shank2b* mRNA was also significantly reduced in heart, liver–gall–spleen, skin–muscle, and gonad, compared to WTs, suggesting a loss of mutant transcript via nonsense-mediated decay (Supplementary Figure S1E).

We observed no difference in gross morphology between WT and homozygous *shank2b^−/−^* adults (Supplementary Tables S6 and S7). While *shank2b^−/−^* zebrafish larvae at 58 h post-fertilization (hpf) displayed a small head phenotype and shorter body length (Supplementary Figure S2A–D) compared to WTs, the ratios of several head measurement parameters to body length were normal (Supplementary Figure S2E,F) and all brain regions were well-preserved in adult mutant fish (Supplementary Figure S2G).

### 2.2. Autism-like Behaviors in Adult and Larval shank2b^−/−^ Zebrafish

Human genetic studies have implicated *SHANK2* in ASDs [15,16,18,21,22,23,24,25], and previous rodent experiments have reported autism-like behaviors in two different lines of *Shank2* mutant mice as well as impaired social and cognitive behaviors in *Shank2*^Δ*e*24^ mice [40,41,42]. We conducted a series of behavioral tests to assess whether *shank2b^−/−^* zebrafish also display ASD-like phenotypes analogous to those associated with *SHANK2* mutations in humans.

**Figure 2:**
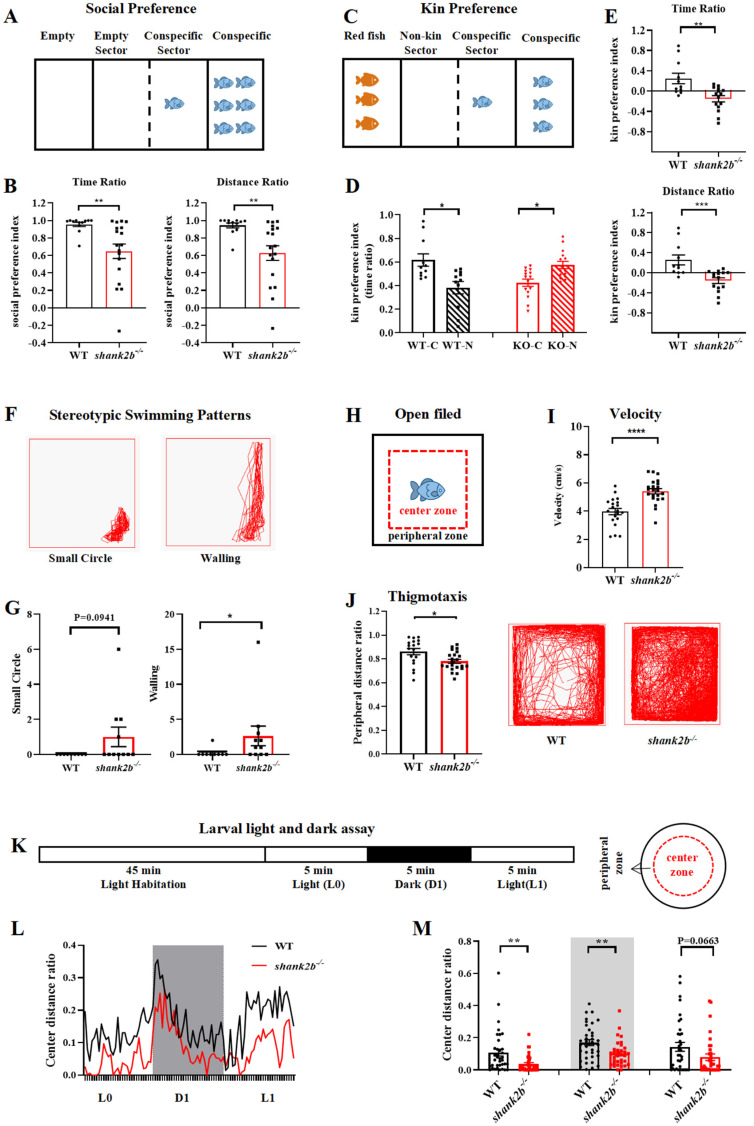
The *shank2b^−/−^* deficient zebrafish displayed autism-like behaviors. (**A**) Schematic diagram of the social preference test of adult male zebrafish. (**B**) The *shank2b^−/−^* zebrafish displayed a significantly reduced preference for conspecifics compared to WT (time ratio, ** *p* = 0.0063; distance ratio, ** *p* = 0.0057; WT, n = 12, *shank2b^−/−^*, n = 18, Student’s *t* test). Data are shown as mean ± SEM. (**C**) Schematic diagram of the kin preference test of adult male zebrafish. (**D**) The *shank2b^−/−^* zebrafish significantly preferred spending time exploring non-kin sector to interact with red fish (n = 15, * *p* = 0.0278, paired *t* test), whereas WT zebrafish preferred conspecifics (n = 11, * *p* = 0.0498, paired *t* test). (**E**) In addition, the mutants displayed a significantly reduced kin preference index compared to WT zebrafish (time ratio, ** *p* = 0.0018; distance ratio, *** *p* = 0.007; Student’s *t* test). Data are shown as mean ± SEM. (**F**) The stereotyped swimming patterns in zebrafish are shown as “small circle” and “walling”. (**G**) The *shank2b^−/−^* zebrafish at 4 mpf exhibited a trend of higher frequency of “small circle” stereotyped behavior (*p* = 0.0941) and significant increase in “walling” stereotyped behavior (* *p* = 0.0284), Mann–Whitney test. (**H**) Schematic diagram of the open field test. (**I**) The velocity of *shank2b* mutant at 2 mpf, quantified by average distance moved per one second over 30 min in this test, was increased compared to that of WT zebrafish (WT n = 20, *shank2b^−/−^* = 22, **** *p* < 0.0001, Student’s *t* test). Data are shown as mean ± SEM. (**J**) Representative traces of individual WT and *shank2b^−/−^* zebrafish at 2 mpf in the thigmotaxis test. The *shank2b^−/−^* zebrafish tended to stay in the center area so that distance ratio of thigmotaxis was decreased significantly (WT n = 18, *shank2b^−/−^* = 22, * *p* = 0.0104, Student’s *t* test). Data are shown as mean ± SEM. (**K**) Light and dark experimental setup for analysis of larvae at 7 days post-fertilization (dpf). (**L**) The horizontal axis denotes the time period of the alternating light and dark conditions. The vertical axis shows the ratio of distance moved in the center ring. (**M**) Under illumination, the *shank2b^−/−^* larvae at 7 dpf exhibited a significant decrease during L0 period and a trend of decrease during L1 period. The results also showed significant decrease under dark condition (D1) compared to WT larvae. L0 (left), ** *p* = 0.0036; D1 (middle), ** *p* = 0.0033; L1, *p* = 0.0663. WT larvae n = 36, *shank2b^−/−^* larvae n = 34. Student’s *t* test. Data are shown as mean ± SEM.

We first tested social preference behaviors using three-chamber tests. As shown in Figure 2B, adult *shank2b^−/−^* mutants in the central chamber spent less time and swam a shorter distance in the conspecific sector compared to WTs. Moreover, mutants showed no preference for kin fish in one outer chamber (i.e., spent no more time swimming in the zone adjacent to the chamber) relative to non-kin fish in the other outer chamber, in contrast to the significant kin preference of WTs (Figure 2D). Furthermore, *shank2b^−/−^* zebrafish spent much less time and swam a shorter distance parallel to conspecifics than WT zebrafish (Figure 2E). These results suggest that shank2b deficiency leads to low social preference in zebrafish.

Also consistent with an ASD-like phenotype, adult *shank2b^−/−^* zebrafish spent more time engaged in repetitive and stereotyped swimming behaviors in the open field, especially stereotypic wall swimming (walling) (Figure 2G). Mutants also exhibited a greater frequency of small circle swimming, although the difference from WTs did not reach statistical significance (Figure 2G). Overall motor activity in the open field was also significantly greater among adult *shank2b^−/−^* zebrafish than WTs (Figure 2I). Moreover, the proportion (%) of total distance traveled in the peripheral zones of a new tank (thigmotaxis) was significantly lower among adult *shank2b^−/−^* fish (Figure 2J), suggesting higher trait anxiety. Similarly, the proportion of swimming distance in the tank periphery relative to the center was greater among zebrafish larvae at 7 days post-fertilization (dpf) compared to age-matched WTs (Figure 2L,M). Larval *shank2b^−/−^* fish also demonstrated a significant decrease in the center distance ratio during the first dark period (D1) of the dark–light cycle compared to WT larvae, as well as a numerical reduction during the first light period (L1). Thigmotaxis was also greater among *shank2b^−/−^* larvae at 7 dpf under continuous illumination (L0). Collectively, these findings suggest that shank2b deficiency leads to an enhanced anxiety-like behavioral phenotype.

### 2.3. Abnormal Responses to Visual and Auditory Stimuli among shank2b^−/−^ Larvae

Humans with *SHANK2* deficiency are reported to have atypical sensory processing [20,22]. To determine if *shank2b* larvae exhibit similar sensory processing abnormalities, we compared visual motor responses (VMRs) to light–dark or dark–light transitions and startle responses to high-intensity sound stimuli between mutants and WT larvae.

**Figure 3:**
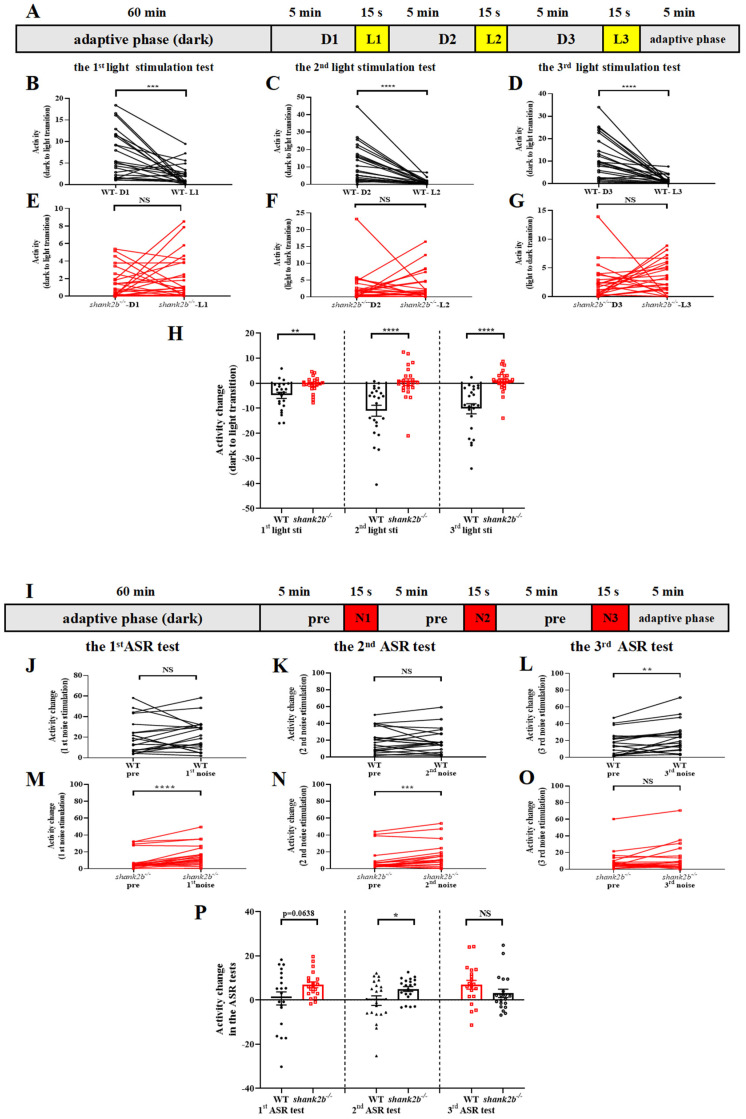
Abnormal response to visual and auditory stimuli in *shank2b* larva. (**A**) Scheme and behavioral setup applied for locomotor activity tracking in VMR response to light stimulation of zebrafish larvae at 13 dpf. The experiment consisted of a 60-min adaptation period under continuous dark and a 20 min 45 s testing period consisting of three VMR. The VMR experiment consisted of 5 min of conditioning to darkness and 15 s of stimulation by a sudden light stimulus. (**B**–**D**) WT larvae at 13 dpf exhibited normal decrease in response to sudden light stimuli (1st light stimuli, *** *p* = 0.0006; 2nd light stimuli, **** *p* < 0.0001; 3rd light stimuli, **** *p* < 0.0001. n = 23, paired *t* test), (**E**–**G**) whereas *shank2b^−/−^* models showed completely different response during dark–light transitions, characterized by dynamic slight increases in activity without statistical significance (1st light stimuli, ns *p* = 0.3395; 2nd light stimuli, ns *p* = 0.7621; 3rd light stimuli, ns *p* = 0.3446. n = 24, paired *t* test). (**H**) Column plots compare activity detected during the 1 min before and the 15 s after each light stimulation exposure between WT and *shank2b* mutants. Each transition from dark to light induced significant difference in activity change between WT and *shank2b* larva (1st light stimuli, ** *p* = 0.0029; 2nd light stimuli, **** *p* < 0.0001; 3rd light stimuli, **** *p* < 0.0001. WT, n = 23, *shank2b^−/−^* n = 24, Student’s *t* test). Data are presented as the mean ± SEM. (**I**) Scheme and behavioral setup applied for locomotor activity tracking in AMR response to loud noise stimulation of zebrafish larvae at 13 dpf. The experiment consisted of a 60 min adaptation period and a 20 min 45 s testing period consisting of three AMR tests. An AMR experiment consisted of 5 min of conditioning to ambient sound and 15 s of stimulation by a sudden loud noise. (**J**–**L**) WT larvae at 13 dpf exhibited a significant and robust increase in activity induced by the third exposure to the sudden noise stimulus after twice being exposed to ASR assays (1st noise stimuli, ns *p* = 0.8148; 2nd noise stimuli, ns *p* = 0.8905; 3rd noise stimuli, ** *p* = 0.0028. n = 20, paired *t* test). (**M**–**O**) The first and second noise stimuli induced significant increase in activity of *shank2b^−/−^* larvae (1st noise stimuli, **** *p* < 0.0001; 2nd noise stimuli, *** *p* = 0.0003; 3rd noise stimuli, ns, *p* = 0.1257. n = 20, paired *t* test). (**P**) Column plots compare activity detected during the 1 min before and the 15 s after each loud noise stimulation exposure between WT and *shank2b* mutants. Similarly, in first two sudden loud noise stimulations, the velocity change of *shank2b* larva at 13 dpf was more dramatic than that of the WT larvae (1st noise stimuli, *p* = 0.0638; 2nd noise stimuli, * *p* = 0.0369; 3rd dark noise, ns *p* = 0.1645. WT n = 20, *shank2b^−/−^* n = 20, Student’s *t* test). Data are presented as the mean ± SEM.

Zebrafish larvae normally demonstrate dramatically enhanced activity in response to an abrupt light-to-dark transition (darkness stimulation) (Supplementary Figure S3B–D) and a brief period of hypoactivity when exposed to a sudden dark-to-light transition (light stimulation) (Figure 3B–D). When exposed to abrupt light stimulation, however, 13 dpf *shank2b^−/−^* larvae showed no significant change in activity, and rather than the expected hypoactivity, some animals exhibited increased activity in response to a second or third dark-to-light transition (Figure 3E–G). Overall, there was a significant difference in activity during dark-to-light transitions between WT and *shank2b^−/−^* mutant larvae (Figure 3H). In contrast, both genotypes exhibited increased activity in response to a light-to-dark transition (Supplementary Figure S3E–G) and there was no significant difference in the magnitude of this change between genotypes over three light-to-dark transition cycles (Supplementary Figure S3H).

Zebrafish larvae normally respond to sudden broad-band noise with a dramatic increase in motor activity (Figure 3J,K), which is analogous to the acoustic startle response (ASR) observed in rodents and humans. Furthermore, in accord with ASRs in other animals, WT larvae at 13 dpf demonstrated a degree of habituation upon repeated stimulus presentation. However, mutants exhibited both markedly enhanced activity compared to WTs in response to the first stimulus, indicating greater baseline ASR sensitivity, as well as a greater response to the second stimulus (Figure 3M,N) compared to WTs (Figure 3L), indicating reduced habituation. The ASR of mutants was also numerically greater than that of WTs in response to a third stimulus, although the difference did not reach statistical significance (Figure 3P).

### 2.4. GABAergic Deficits in shank2b^−/−^ Zebrafish

**Figure 4:**
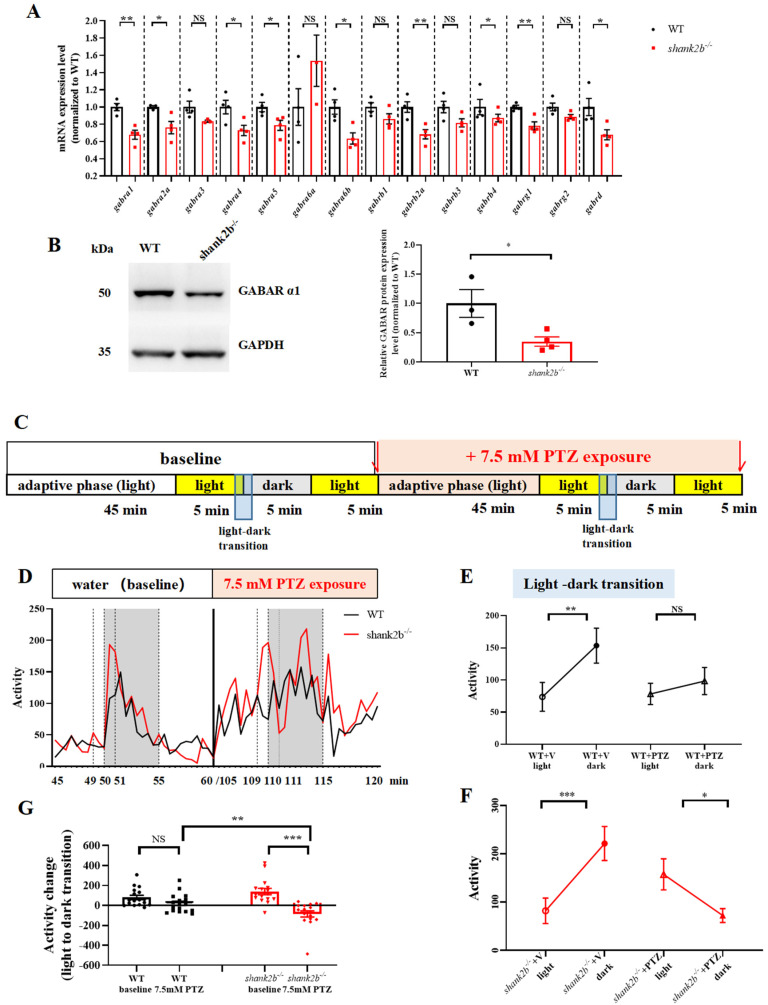
The *shank2b* mutants display GABAergic deficits. (**A**) RT-qPCR showed altered expression levels of GABAR subunits in adult *shank2b^−/−^* brain tissue. *gabra1*, ** *p* = 0.003; *gabra2a*, * *p* = 0.0394; *gabra3* ns *p* = 0.0993; *gabra3* ns *p* = 0.0660; *gabra4*, * *p* = 0.0341; *gabra5*, * *p* = 0.0371; *gabrg2* ns *p* = 0.0730; *gabra6a* ns *p* = 0.3488; *gabra6b*, * *p* = 0.0138; *gabrb1* ns *p* = 0.1258; *gabrb2a*, ** *p* = 0.0083; *gabrg1*, ** *p* = 0.005; *gabrd*, * *p* = 0.0318; n = 3–4 for each group. Student’s *t* test. Data are presented as the mean ± SEM. (**B**) Western blot analysis of GABA _A_ receptor α1 protein in adult *shank2b^−/−^* brain tissue. * *p* = 0.0313, WT n = 3, *shank2b^−/−^* n = 4, Student’s *t* test. Data are presented as the mean ± SEM. (**C**) Pentylenetetrazol (PTZ)-treated behavior experimental procedure. (**D**) The experiment consisted of basal activity change during light to dark transition and 7.5 mM PTZ-treated induced activity change exposure to the same basal experiment. (**E**) WT larva at 9 dpf exhibited a dynamic increase of activity during light–dark transitions (WT + v (vector) light vs. WT + v dark, ** *p* = 0.0022,; n = 17, paired *t* test). Remarkably, PTZ concentrations of 7.5 mM of WT larva at 9 dpf did not elict a decline in activity (WT + PTZ light vs. WT + PTZ dark, ns, *p* = 0.3869, n = 17, paired *t* test). (**F**) However, 7.5 mM PTZ elicited a decline in activity of *shank2b* mutants (*shank2b^−/−^* + V light vs. *shank2b^−/−^* + V dark, *** *p* = 0.0005; *shank2b^−/−^* + PTZ light vs. *shank2b^−/−^* + PTZ dark, * *p* = 0.0157; n = 16, paired *t* test). (**G**) Interestingly, the activity change of *shank2b* larvae at 9 dpf after 7.5 mM PTZ treatment was more than the activity change before PTZ treatment (*shank2b^−/−^* − baseline vs. *shank2b^−/−^* + PTZ, *** *p* = 0.0006, n = 16, paired *t* test), and even greater than the activity change after WT larvae treated with PTZ (WT + PTZ vs. *shank2b^−/−^* + PTZ, ** *p* = 0.0096, WT + PTZ n = 17, *shank2b^−/−^* + PTZ n = 16 Student’s *t* test). Remarkably, WT larva at 9 dpf did not have significant difference in the activity change during light–dark transition before and after 7.5 mM PTZ treatment (WT-baseline vs. WT + PTZ, ns *p* = 0.0961, n = 17, paired *t* test).

Atypical sensory responses in ASD mouse models are associated with alterations in GABAergic interneuron development [31,43,44]. To examine if alterations in GABAergic neuron development or signaling also occur in *shank2b* mutants, we compared the mRNA levels of GABA receptor subunits between adult WT and *shank2b^−/−^* mutants. Indeed, RT-qPCR revealed significant decreases in the mRNA expression levels of *gabra1*, *gabra2a*, *gabra4*, *gabra5*, *gabra6b*, *gabrb2a*, *gabrg1*, and *gabrd* in *shank2b^−/−^* zebrafish compared to WTs (Figure 4A). Moreover, GABA_A_ receptor α1 protein expression was also significantly reduced in mutants, suggesting GABAergic system dysfunction and a concomitant excitatory shift in the excitation/inhibition balance (Figure 4B). To provide further evidence for such a shift, we examined the susceptibility of WT and mutant larvae to seizures induced by the GABA_A_ receptor antagonist pentylenetetrazol (PTZ), which is widely used to induce seizures in both rodent models and zebrafish. PTZ-induced seizures appear as suppression in the expected motor activity increase following a sudden transition from light to darkness. Consistent with an excitatory shift in excitation/inhibition balance due to deficient GABA receptor signaling, *shank2b* mutants demonstrated greater sensitivity to PTZ-induced behavior changes (Supplementary Figure S4B–D,H,I). Moreover, PTZ treatment induced significantly more activity change in *shank2b^−/−^* larva at 9 dpf compared to age-matched WT larvae during the light–dark transition (Figure 4D–G). Collectively, our findings provide strong evidence that neurodevelopmental GABAergic signaling deficits underlie the ASD-like behavioral abnormalities in *shank2b* mutants.

## 3. Discussion

Human genetic studies have implicated *SHANK2* in a wide spectrum of neurodevelopmental conditions, including autism symptoms, intellectual disability, hyperactivity, and anxiety. Two out of three patients in our clinical cohort screen harboring *SHANK2* mutations also exhibited hyper-sensitivity to visual, auditory, and tactile stimuli, but there have been few descriptions of the *SHANK2*-deficient sensory phenotype. Aberrant sensory reactivity is now regarded as a diagnostic feature of ASDs, but only one of the previous 18 patients known to harbor de novo *SHANK2* variants has received detailed examinations of atypical sensory processing. The current study expands the spectrum of potential behavioral phenotypes exhibited by *shank2*-deficient animal models. Furthermore, this phenotypic profile is generally consistent with the spectrum of clinical features ascribed to *SHANK2*-related disorders in humans. These *shank2b* mutant zebrafish were hyperactive in the open field and displayed an anxiety-like phenotype under continuous illumination, reduced social preference and kin preference, and greater stereotypy. Further, *shank2b* mutants showed reduced expression levels of GABA receptor subunit mRNAs and α1 subunit protein, and increased sensitivity to drug-induced seizures. Intriguingly, several molecules involved in GABAergic signaling have been implicated in autistic sensory symptoms [11]. Taken together, our data suggest that *shank2b^−/−^* zebrafish are a valuable model for dissecting the pathophysiology of ASD in humans, and that the sensory abnormalities of ASD may stem at least in part from reduced GABA receptor activity.

Studies have shown that two lines of *Shank2* knockout mice altering different regions of the protein displayed similar autism behavioral phenotypes [40,41], which were different from bipolar-associated mania-like behavior exhibited in the third line of *Shank2*^Δ*ex*24^ knockout mice [42]. Generally, all three conventional and other conditional *Shank2*-deficient mouse models showed distinct neuropsychiatric-like phenotypes, having no sensory abnormalities except hyposensitivity to painful stimuli. Here, we created the *shank2b^−/−^* model in zebrafish to test the common correlation of autism and sensory processing problems. Potential sensory processing deficits associated with *shank2b* deficiency were examined in detail using two light-stimulation-based tasks and one noise-stimulation-based task, all of which revealed enhanced stimulus sensitivity in mutant larvae compared to WT larvae. Mutants exhibited an exaggerated acoustic startle response and reduced ASR habituation as well as relative hyperactivity in response to sudden transitions from darkness to light. Our current behavioral study suggests that this visual response hyperactivity contributes to reduced kin preference. Similarly, visual hypersensitivity has been observed in several mouse models of ASD and related neurodevelopmental disorders, including lines with mutations/deficiencies in *Mecp2*, *Fmr1*, *Shank3*, *Gabrb3*, and *Cntnap2* [31,43,45,46,47,48]. These abnormal sensory behaviors support the face validity of the zebrafish model for studying *SHANK2*-related ASD in humans. 

These ASD-related sensory abnormalities and other symptomatic behaviors may arise from an imbalance between synaptic excitation and inhibition [28] due to insufficient inhibitory GABA neurotransmission [49]. In turn, GABAergic dysfunction may be a downstream consequence of mutations in genes essential for synaptic formation, signaling, and molecular organization [49], as several mouse lines harboring putative ASD mutations show disrupted inhibitory signaling. For example, deletion of the autism-risk gene contactin-associated protein 2 (*Cntnap2*) reduced the number of GABAergic interneurons in mouse cortex, including fast-spiking parvalbumin-immunopositive interneurons [50]. Zebrafish *cntnap2ab* mutants also exhibited reductions in GABAergic neuron numbers, particularly of parvalbumin-positive (PV+) interneurons [51]. In mice lacking Shank3, PV+ interneurons were decreased in the somatosensory cortex (S1) and basolateral amygdala (BLA) [45]. While Shank family proteins are enriched in the excitatory postsynaptic density, numerous studies also suggest widespread expression in GABAergic neurons, including PV-positive interneurons [52,53,54]. For instance, PV immunoreactivity was also reduced in *Shank1* knockout mice [55] and GABAergic neurotransmission was impaired in *Shank2* knockout mice [56].

Interneurons are critically involved in sensory processing [54], and the aforementioned associations between *shank* gene deficiencies and GABAergic dysfunction in the CNS suggest that *shank2* and other family members act as part of a biological network regulating GABAergic signaling in multiple sensory systems, leading to the sensory processing abnormalities observed in ASD. In our study, not only did these mutant fish display sensory phenotypes consistent with humans, they also had decreased expression levels of GABA receptor as well as increased sensitivity to PTZ-induced seizures. RT-qPCR analysis revealed reduced mRNA expression levels of subunit genes *gabra1*, *gabra2a*, *gabra4*, *gabra5*, *gabra6b*, *gabrb2a, gabrg1*, and *gabrd*, and multiple GABA_A_ receptor subunits, including GABRG1, GABRA2, GABRA4, and GABRA5 have been linked to autism by genetic analyses [57,58,59]. Expression levels of GABA_A_ receptor α1 protein, which plays a key role in defining the critical period for plasticity in developing rodent visual cortex [60], was also significantly reduced in *shank2b*^−/−^ zebrafish. These GABA_A_ receptor gene and protein deficits were associated with hyperactivity under illumination as well as increased sensitivity to PTZ-induced seizures, suggesting loss of inhibitory drive in mutant zebrafish brain. GABA_A_ receptor activity is critical for neurodevelopment by stimulating neural progenitor cell proliferation, migration, and differentiation, neurite growth, and synaptogenesis [61]. Furthermore, GABAergic interneurons help establish the receptive field organization of sensory cortex. Moreover, there is now strong convergent evidence for abnormal expression patterns of GABA_A_ receptor genes and other genes expressed at relatively high levels by GABA interneurons in idiopathic ASD [49,62]. Thus, we suggest that *shank2* contributes to GABAergic network development in zebrafish, and that loss of *shank2* expression during development results in aberrant sensory system organization and sensory processing throughout life, which in turn contributes to other ASD-like behavioral phenotype.

The duplicated and conserved shank2a and shank2b are similar and share high sequence identity at the overall protein structure (Supplementary Table S5), whereas a loss of an evolutionary conserved domain of shank2a can be detected (Supplementary Figures S5 and S6). In contrast, several important protein-ANK, PDZ and SAM C-terminal region, of human SHANK2 are more highly conserved in shank2b than shank2a (Supplementary Tables S3 and S4). Therefore, *shank2b* is more suitable as a model for human *SHANK2*. However, based on the expression of *shank2a* and *shank2b* transcripts in the brain of zebrafish, we also speculate that both paralogs may be important for the formation of the nervous system in zebrafish. It would be interesting to compare the phenotypes of *shank2a^−/−^*, *shank2b^−/−^* and *shank3&b* double homozygous models in parallel. Another limitation of this study is that we did not directly analyze GABAergic interneuron development or examine whether GABA agonists mitigate the behavioral abnormalities of *shank2b* mutants. Moreover, *shank3&b* deficient zebrafish model should be more appropriate to fully unravel the molecular mechanisms by which *shank2* regulates GABAergic network development and behavior in zebrafish.

**Figure 5:**
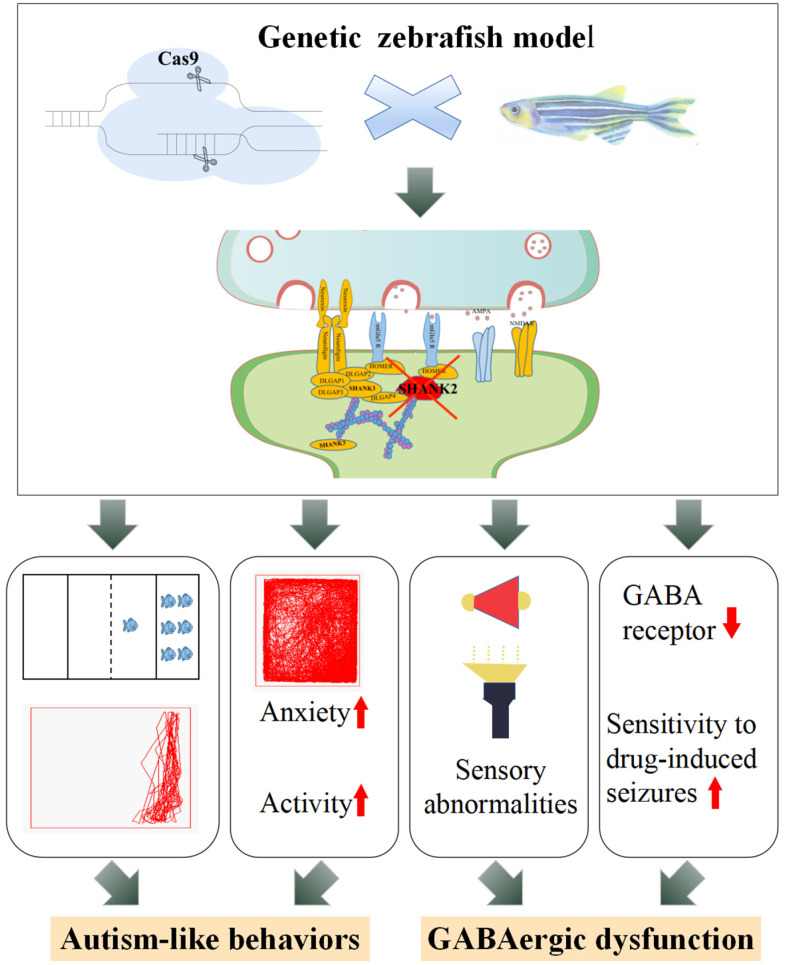
Potential mechanisms for regulation of GABAergic system in the *shank2*-deficient zebrafish model.

In conclusion, we revealed ASD-like behavioral and sensory deficits associated with GABAergic system dysfunction in a novel *shank2b*-deficient zebrafish model (Figure 5). This model is a valuable tool for dissecting the pathophysiology of *SHANK2*-related disorders in humans and may assist in the development of new treatments for ASD with abnormal sensory processing.

## 4. Materials and Methods

### 4.1. Fish Maintenance and Husbandry

Zebrafish of the Tubingen (Tu) strain were housed at the Institute of Zebrafish, Children’s Hospital of Fudan University of China (Shanghai, China). Both larvae and adults were maintained in a recirculation system at 28.5 °C under a 14 h/10 h light/dark circadian cycle. All animal experiments were performed in compliance with the Guiding Principles for the Care and Use of Laboratory Animals and received approval from the institutional animal care committee of the Children’s Hospital of Fudan University.

### 4.2. Generation of shank2b-Deficient Zebrafish Using the CRISPR-Cas9 System

The zebrafish *shank2b* gene and exon/intron boundaries were identified by searching the NCBI database (gene ID: *shank2b* NC_007136.7), and a mutation in *shank2b* was generated using CRISPR/Cas9 editing as previously reported [63]. The site-specific single guide RNA (sgRNA) of *shank2b* was designed to target the 10th exon sequence 5′-GGATCGGAGCAGCACTCGCG-3′ and synthesized by in vitro transcription using the MAXIscript™ T7 kit (AM1314M, Invitrogen, Waltham, MA, USA). A micro-injector was then used to inject 150: 600 pg of sgRNA: cas9 Nuclease (En Gen^TM^ spy cas9 NLS #M0646, New England Biolabs, Ipswich, MA, USA) into one-cell-stage fertilized WT zebrafish embryos (*F_0_*). Injected embryos were raised for 72 h before preparing genomic DNA to check mutagenic efficiency using the primers listed in Supplementary Table S1 and Sanger sequencing. Identified mutations were then analyzed for unique restriction digest sites usable for genotyping. Progeny were propagated via a series of out-crossings with WT zebrafish and genotyping of each generation. Eventually, these animals were in-crossed to obtain the homozygous deficient *shank2b^−/−^* mutants.

### 4.3. RT-qPCR

Changes in gene expression during development were assessed by RT-qPCR analysis of embryos at 24 hpf, 48 hpf, 3 dpf, and 5 dpf. All analyses included three or four independent embryo pools with 25 embryos per pool. In addition, brains were collected from larvae at 3 weeks post-fertilization (wpf) and adults at 2 months post-fertilization (mpf) (three independent brain pools, 10 fish per pool). Brain, heart, liver–gall–spleen, skin–muscle, and spermatic cord tissues were also collected from adult male zebrafish and ovaries from adult female zebrafish at 4.5 mpf (three pools per tissue type, 5 fish per pool), and quick-frozen on dry ice. Total RNA was extracted from harvested tissues and whole embryos using the MiniBEST Universal RNA Extraction kit (No. 9767, Takara, Japan) according to the manufacturer’s protocol and reverse transcribed to cDNA using the PrimeScript™ RT reagent Kit with gDNA Eraser (Perfect Real Time) (RR047A, Takara, Japan) following the manufacturer’s instructions. RT-qPCR was performed using an Applied Biosystems™ 7500 system (Bio-Rad, USA) and TB Green Premix Ex Taq II (Tli RNaseH Plus) (RR820A, Takara, Japan) to estimate the mRNA expression levels of *shank2b*, *shank2a*, and GABA receptor subunits according to the manufacturers’ protocols. Expression levels were normalized to that of *Rpl13α* as the internal control using the delta CT method. The primer sequences for RT-qPCR are listed in Supplementary Table S1. All measurements were conducted on at least three independently harvested pools of tissue or whole embryos.

### 4.4. Western Blot

A custom antibody-recognizing zebrafish shank2a and shank2b (RB1661) was generated by HUABIO using a peptide antigen shared by both proteins (CRSLSMPDTSEDIPP-amide, corresponding to amino acids 860 to 873 of shank2). For Western blotting, total protein was isolated from the brains of 4.5 mpf WT and *shank2b^−/−^* zebrafish, separated by SDS-PAGE (50 μg per gel lane), and transferred to polyvinylidene difluoride membranes. Membranes were blocked with 5% BSA or 7% non-fat skim milk powder at room temperature for 2 h, incubated with an affinity-purified primary antibody against GABARα (Abcam, ab211131, 1:1000) overnight at 4 °C, washed in TBS containing 0.1% Tween-20 (TBST), and incubated with horseradish peroxidase (HRP)-conjugated secondary antibodies (1:5000) for 1 h at room temperature. Following three washes in TBST, the blots were incubated with ECL reagent (BeyoECL Plus, P0018M) and exposed to Kodak X-ray film (Tanon 5200). The gray scale values of proteins were analyzed by ImageJ software (NIH, Bethesda, MD, USA), and normalized to GAPDH (1:5000) as the gel-loading control.

### 4.5. Larval Responses to Light and Darkness

Larval zebrafish at 7 dpf were placed individually into the wells of 24-well plates for video recording of activity using the Zebrabox system (ViewPoint Life Sciences, Lyons, France). Every larva was given 45 min to habituate to the illumination environment before video acquisition. Subsequently, larvae were exposed to 5 min of continuous illumination, followed by one dark/light cycle (5 min of dark and 5 min of light, Figure 2K). The light intensity was 100% in the light period and 0% in the dark period. The time and distance traveled were recorded every 30 s using the Viewpoint tracking system and custom software. The total recording time was 60 min. For analysis, a circle was drawn at the center of each well that divided the well into inner and outer regions of equal area (Figure 2K) and was thigmotaxis defined as,

$$\mathrm{Larval}\;\mathrm{Thigmotaxis}=\frac{\mathrm{Distance}\;\mathrm{moved}\;\mathrm{in}\;\mathrm{the}\;\mathrm{center}\;\mathrm{zone}}{\;\mathrm{Distance}\;\mathrm{moved}\;\mathrm{in}\;\mathrm{the}\;\mathrm{whole}\;\mathrm{zone}}$$

### 4.6. Acoustic Startle Response

Wild type and *shank2b^−/−^* larvae were gathered at 13 dpf to evaluate the ASR following a previously published method [64]. Briefly, larvae were placed individually in wells of 24-well plates and exposed to broad-band noise within a ZebraBox equipped with an infrared illuminator for imaging in the dark. The broad-band noise was computer generated and played through two commercial loudspeakers placed inside but not physically connected to the chamber. Video footage was shot at 25 frames per second (fps) and binned into 1-s time windows to evaluate the ASR. After a 60 min acclimatization period, zebrafish were monitored during three individual ASR experiments which consisted of 5 min conditioning to ambient sound and 15 s of stimulation by a sudden loud noise (Figure 3I). The activity counts in groups during the 1 min before and the 15 s during each sudden sound exposure were quantified and used to calculate the ASR according to the equation

Activity change in the ASR tests = Average activity during 15 s-noise phase (post) − Average activity during 60 s-pre-noise (pre)

### 4.7. Visual Motor Behavior Assay

Larvae at 13 dpf were pipetted individually into the wells of 24-well plates and visual motor behavior analyzed during light-to-dark and dark-to-light transitions. Briefly, the specifications for video acquisition and tracking were the same as those in the ASR assay. For analysis of behavioral changes during light-to-dark transitions, fish were acclimated to the observation chamber under continuous maximum white light illumination for one hour (adaptive phase). Then, three illumination-off cycles (5 min illumination, followed by darkness) were delivered (Supplementary Figure S3A). For analysis of behavioral changes during dark-to-light transitions, fish were acclimated to the observation chamber under darkness for 1 h (adaptive phase). Then, three illumination-on cycles (5 min darkness, followed by 15 s light) were delivered (Figure 3A).

The activity changes were analyzed for both WT and *shank2b* mutants according to the equations

Activity change (light to dark transition) = Activity detected in post dark 15 s − Activity detected in pre illumination 60 s

Activity change (dark to light transition) = Activity detected in post illumination 15 s − Activity detected in pre dark 60 s

### 4.8. Pentylenetetrazol Activity Assay

To assess behavioral alterations and seizure susceptibility as indices of an E/I imbalance, we measured the dose–response of 9 dpf larvae to the GABA_A_ receptor antagonist PTZ. Wild type and *shank2b^−/−^* larvae were placed individually in wells of 24-well plates containing standard embryo water plus different concentrations of PTZ (P6500, Sigma-Aldrich, Zwijndrecht, The Netherlands) pre-dissolved in distilled water. Briefly, four treatment groups were established: WT-standard water, WT-PTZ, *shank2b^−/−^*-standard water, and *shank2b^−/−^*-PTZ. The PTZ was first dissolved in distilled water and then further in methylene blue diluted system water (0.15 mg/L methylene blue, 8.01 mg/L NaCl, 5.04 mg/L KCl, 5.50 mg/L Na_2_HPO_4_, 0.44 mg/L KH_2_PO_4_, 1.30 mg/L CaCl_2_, 1.00 mg/L MgSO_4_, and 4.20 mg/L NaHCO_3_) to yield working solutions. Subsequently, 500 μL of PTZ working solution was quickly added to 500 μL standard egg water per well to reach a final volume of 1 mL/well containing 1, 2.5, 5, or 7.5 mM PTZ. The plates were then placed in a custom-modified Zebrabox to record video of zebrafish larval activity. After a 5 min acclimation period with illumination, spontaneous activity was recorded for 45 min. Animals were then exposed to one 10 min light–dark cycle (5 min illumination followed by 5 min dark) to examine responses to changes in lighting conditions under the influence of PTZ. Responses were analyzed by comparing the distance traveled in the one min before and after transition from light to darkness. Each experimental session lasted for 60 min in total, including the acclimation period (Supplementary Figure S4A).

We used a novel behavioral test to determine whether 7.5 mM PTZ, selected because it induced abnormal activity in *shank2b* mutants, elicited the greatest differential response in WT versus mutant fish at 9 dpf. Briefly, *shank2b^−/−^* and WT larvae at 9 dpf were pipetted individually into the wells of 24-well plates containing 1 mL of standard embryo water and activity recorded using the paradigm described above. Subsequently, 500 μL of egg water was removed and 500 μL of PTZ in egg water was added to the well to yield a final PTZ concentration of 7.5 mM. We used a repeated light–dark challenge assay to elicit PTZ-induced responses (Figure 4C). Responses were analyzed by normalizing the post-PTZ activity of each WT and *shank2b* mutant fish to baseline.

### 4.9. Open Field Test

The locomotor activity of adult zebrafish was tested at 2–4 mpf in an adapted open field paradigm (Figure 2H). Videos were captured in a 30 × 30 × 30 cm opaque tank filled with system water using a suspended camera. Each male zebrafish was habituated in the tank for 5 min before the 30 min recording period. Time in motion and distance traveled were analyzed every 30 s using Zebralab software (ViewPoint Life Sciences, Lissieu, Calvados, Lower Normandy Region, Lyons, France). For analysis of stereotyped behaviors, an experimenter blind to genotype and treatment history scored the swimming pattern each minute from the transformational visual route of fish trajectory, and counted the frequency of two stereotyped swimming patterns, small circling and walling (Figure 2F).

The locomotor activity of adult zebrafish was tested at 2–4 mpf in an open field paradigm (Figure 2H). Videos were captured in a 30 × 30 × 30 cm opaque tank filled with system water using a suspended camera right above. Each male zebrafish was habituated in the tank for 5 min before the 30 min test. The time and distances were collected and analyzed every 30 s from the 30 min recorded video using Zebralab software. For stereotyped behaviors, the experimenter was required, under double-blind control, to score the swimming pattern within each minute of the Zebralab-software-generated visual route of fish trajectory, and count the number of the four stereotyped swimming pattern episodes separately, specifically, small circling and walling (Figure 2F).

Thigmotaxis, a well-validated sign of anxiety in adult zebrafish, was scored as

$$\mathrm{Thigmotaxis}=\frac{\mathrm{Distance}\;\mathrm{moved}\;\mathrm{in}\;\mathrm{the}\;\mathrm{peripheral}\;\mathrm{zone}}{\;\mathrm{Distance}\;\mathrm{moved}\;\mathrm{in}\;\mathrm{the}\;\mathrm{whole}\;\mathrm{zone}}$$

### 4.10. Three-Chamber Social Preference Tests

Social preference was tested in a transparent mating tank (dimensions 21 × 11 × 7.5 cm) separated into three chambers by two transparent dividers. Six male WT conspecifics of similar size were placed into one outer chamber while the other outer chamber was left unoccupied (Figure 2A). Then, the subject male zebrafish (WT or mutant) at 3.5 mpf was placed into the middle chamber and given five minutes of free access to the entire apparatus. Videos were recorded for 30 min. Social preference behavior was quantified as the distance or time spent adjacent to the group of conspecifics. The distance ratio was the distance traveled in the conspecific sector adjacent to the conspecific chamber divided by the total distance traveled, and the time ratio was the time spent in the conspecific sector adjacent to the conspecific chamber divided by the total test time.

To further assay the sociality of the *shank2*^−/−^ zebrafish, we performed a kin preference test in the same tank. Briefly, three adult kin zebrafish were placed in the left outer chamber and three adult non-kin zebrafish were placed in the right outer chamber (Figure 2C). Then, the subject male zebrafish (WT or mutant) at 3.5 mpf was placed into the middle chamber. Social preference was assessed by a social preference index (SPI) where

$$\mathrm{SPI}=\frac{\mathrm{Distance}/\mathrm{time}\;\mathrm{in}\;\mathrm{conspecific}\;\sec\mathrm{tor}\;-\;\mathrm{Distance}/\mathrm{time}\;\mathrm{in}\;\mathrm{empty}\;\sec\mathrm{tor}/\mathrm{non}\;\mathrm{kin}\;\sec\mathrm{tor}}{\;\mathrm{Distance}/\mathrm{time}\;\mathrm{in}\;\mathrm{both}\;\sec\mathrm{tors}}$$

### 4.11. Statistical Analysis

All data were processed using the GraphPad Prism Software (GraphPad Software, San Diego, CA, USA). Significance of differences were determined either with one-tailed Student’s *t* test, Mann–Whitney test, or paired *t* test, as indicated.

## Data Availability

The datasets used and/or analyzed during the current study are available from the corresponding author upon reasonable request.

## Supplementary Materials

The following supporting information can be downloaded at: https://www.mdpi.com/article/10.3390/ijms24032208/s1.
