# Supplementary material for: Behavioral and Sensory Deficits Associated with Dysfunction of GABAergic System in a Novel shank2-Deficient Zebrafish Model
Source: Int J Mol Sci. 2023 Jan 22;24(3):2208. doi: 10.3390/ijms24032208 (PMC9916955; doi:10.3390/ijms24032208)
Supplement: Supplementary file 1 [file ijms-24-02208-s001.zip › ijms-2089296-supplementary.pdf]

Supplementary

Behavioral and Sensory Deficits Associated with Dysfunction of GABAergic System in a novel *shank2*-Deficient Zebrafish Model

Yi Wang [1], Chunxue Liu [1], Jingxin Deng [1], Qiong Xu [1], Jia Lin [2], Huiping Li [1], Meixin Hu [1], Chunchun Hu [1], Qiang Li [2] and Xiu Xu [1,*]

[1] Division of Child Health Care, Children's Hospital of Fudan University, National Children's Medical Center, 399 Wanyuan Road, Shanghai 201102, China

[2] Center for Translational Medicine, Institute of Pediatrics, Shanghai key Laboratory of Birth Defect, Children's Hopstial of Fudan University, National Children's Medical Center, 399 Wangyuan Road, Shanghai 201102, China

* Correspondence: Xiu Xu: xuxiu@shmu.edu.cn

Supplementary Table S1: gRNA gene-target sequences, primers for PCR genotyping and RT-qPCR used in this study.

Supplementary Table S2: Protein homology analysis of zebrafish shank2a, shank2b and human SHANK2.

Supplementary Table S3: Domains homology analysis of zebrafish shank2a and human SHANK2.

Supplementary Table S4: Domains homology analysis of zebrafish shank2b and human SHANK2.

Supplementary Table S5: Homology comparison between zebrafish shank2a and shank2b

Supplementary Table S6: Phenotypic characteristics of zebrafish embryos at 24 hpf.

Supplementary Table S7: Phenotypic characteristics of zebrafish embryos at 3 dpf.

Supplementary Figure S1: *shank2* orthologues are conserved in zebrafish and CRISPR-Cas9 induced *shank2b* target-mutation

Supplementary Figure S2: Morphological analysis of *shank2b*$^{-/-}$ zebrafish

Supplementary Figure S3: *shank2b* mutants exhibited normal VMR response to darkness stimuli

Supplementary Figure S4: Dose-response paired plots showing the change in activity during light to dark transition per fish of wild-type or *shank2b*$^{-/-}$ dish at 9dpf exposed to water or increasing concentrations of Pentylenetetrazol (PTZ) for 1 h

Supplementary Figure S5: Different isoforms of zebrafish *shank2a*

Supplementary Figure S6: Different isoforms of zebrafish *shank2b*

Supplementary Table S1. gRNA gene-target sequences, primers for PCR genotyping and RT-qPCR used in this study

Primer	Sequence (5'-3')	Gene ID
shank2b-gRNA target site-E10	GGATCGGAGCAGCACTCGCG	NC_07136.7
shank2b-genotyping PCR-Intron9-F	TGTATGCAGAAATGAAGCTGAAGTGTG	NC_07136.7
shank2b-genotyping PCR-E10/I10-R	AATGTGGGAAGATCACTGACCTGC	
shank2b-qPCR-E10-F	GCCCCGCGAGTGCTGCTC	NM_001128347.1
shank2b-qPCR-E10-R	CCATGGTTTTCACCGTGCCA	
shank2a-qPCR-E2/3-F	ATCGGATCAAGAGAATGGATGGCT	ENSDART00000159950.2
shank2a-qPCR-E3-R	TGAAACACTGCTCTGCGTTCCC	
gabra1-qPCR-F	GGAGAGCGTGTAACCGAAGTCAAG	NM_001077326.1
gabra1-qPCR-R	TTGTTGAGACGGAGCACGGC	
gabar2a-qPCR-F	TTCTCACGGATTTCTAATCCACCTG	XM_009307207.3
gabar2a-qPCR-R	TCTGTCACTCGATCTCCAAGACCA	
gabra3-qPCR-F	TGAAGTTCGGGAGCTATGCCTACA	XM_021469255.1
gabra3-qPCR-R	TGGTCTCTTTGCCGATGACGTG	
gabra4-qPCR-F	TGTCTTTGGAATCACCACCGTCC	NM_001017822.1
gabra4-qPCR-R	GCGTTGGTAAAGTAGTTGACGGCC	
gabra5-qPCR-F	TTTGACCTGCCATCTGAGCCTCT	XM_005166083.4
gabra5-qPCR-R	TGGTCCAAAGCTGGTAACAAAGATG	
gabra6a-qPCR-F	TTGGAAGCTATGCTTACACGAATCG	NM_200731.1
gabra6a-qPCR-R	TGGACGACCTGGACAGAATACAGTC	
gabra6b-qPCR-F	TGACCACTCCCAACAAGCTGTTC	XM_002667357.6
gabra6b-qPCR-R	TCACCTGTGTTTGACTTCAACCTTTC	
gabrb1-qPCR-F	CTCAGGATAACGACTACCGCTGCA	XM_021480764.1
gabrb1-qPCR-R	CGGCAGCTCGATGTTGTCCA	
gabrb2a-qPCR-F	ATTAAGGATCACCACCACCGCTG	NM_001024387.2
gabrb2a-qPCR-R	GCAGTTCGATTCTCTCCACCCC	
gabrb3-qPCR-F	TGACACAAGCGCCAATGAACC	XM_021471811.1
gabrb3-qPCR-R	ACCTCAGACACCATGTCTATGCTCG	
gabrb4-qPCR-F	ACTTTGGAGGTCCTCCAGTCATCG	XM_017353011.2
gabrb4-qPCR-R	GCCACACGGTTGTCCAAAGTCA	
gabrg1-qPCR-F	TCATTAGGCATCACAACTGTGCTCA	XM_009307208.3
gabrg1-qPCR-R	GCTTGTGAAATAATGCAGAGTTCC	

	G	
gabrg2-qPCR-F	CACTTTAAGGTTGACCATTGACGCA	NM_001256250.1
gabrg2-qPCR-R	CGGATATCTCCAACTTCCACAGAGC	
gabrd-qPCR-F	TCAGAAGCCAACATGGAATACACCA	XM_695007.8
gabrd-qPCR-R	TTCTCCACCGTCACGTCATGAAA	
RPL13A(60s)-qPCR-F	TCTGGAGGACTGTAAGAGGTATGC	ENSDART00000023156
RPL13A(60s)-qPCR-R	AGACGCACAATCTTGAGAGCAG	

Supplementary Table S2. Protein homology analysis of zebrafish shank2a, shank2b and human SHANK2

Gene	NCBI reference mRNA number	NCBI reference protein number	Amino acid	vs. human SHANK2 protein (NP_036441.2)	
				Query cover (%)	Identity (%)
shank2a	XM_021467943.1	XP_021323618.1	1707	100%	46.63%
shank2b	NM_001128347.1	NP_001121819.1	1800	100%	60.58%

Supplementary Table S3. Domains homology analysis of zebrafish shank2a and human SHANK2

shank2a domains	NCBI reference protein number	vs. human SHANK2 protein domains (NP_036441.2)	
		Query cover (%)	Identity (%)
ANK	XP_021323618.1	94%	74.23%
PDZ		100%	82.80%
SAM		100%	75.38%

Supplementary Table S4. Domains homology analysis of zebrafish shank2b and human SHANK2

shank2b domains	NCBI reference protein number	vs. human SHANK2 protein domains (NP_036441.2)	
		Query cover (%)	Identity (%)
ANK	NP_001121819.1	100%	80.50%
SH3		100%	76.92%
PDZ		100%	92.55%
SAM		100%	88.06%

Supplementary Table S5 Homology comparison between zebrafish shank2a and shank2b

	NCBI reference protein number	shank2a vs shank2b	
		Query cover (%)	Identity (%)
shank2a	NP_001121819.1	100%	49%
shank2b	XP_021323618.1	-	-
ANK		85%	71%
SH3		-	-
PDZ		100%	83%

SAM	100%	79%

Supplementary Table S6. Phenotypic characteristics of zebrafish embryos at 24 hpf

Genotype	Developmental delay	Death	Normal	Total
shank2b$^{+/+}$(WT)	1 (1.4%)	19 (26.8%)	51 (71.8%)	71 (100%)
shank2b$^{+/-}$	0 (0%)	8 (12.5%)	56 (87.5%)	64 (100%)
shank2b$^{-/-}$	0 (0%)	22 (28.9%)	54 (71.1%)	76 (100%)
Total	1 (0.5%)	49 (23.2%)	161 (76.3%)	211 (100%)

Supplementary Table S5: Person Chi-squared Test, P = 0.088.

Supplementary Table S7. Phenotypic characteristics of zebrafish embryos at 3 dpf

Genotype	Developmental delay	Death	Normal	Total
shank2b$^{+/+}$(WT)	1 (1.4%)	19 (26.8%)	51 (71.8%)	71 (100%)
shank2b$^{+/-}$	0 (0%)	8 (12.5%)	56 (87.5%)	64 (100%)
shank2b$^{-/-}$	0 (0%)	22 (28.9%)	54 (71.1%)	76 (100%)
Total	1 (0.5%)	49 (23.2%)	161 (76.3%)	211 (100%)

Supplementary Table S6: Person Chi-squared Test, P = 0.088.

Supplementary Figure S1

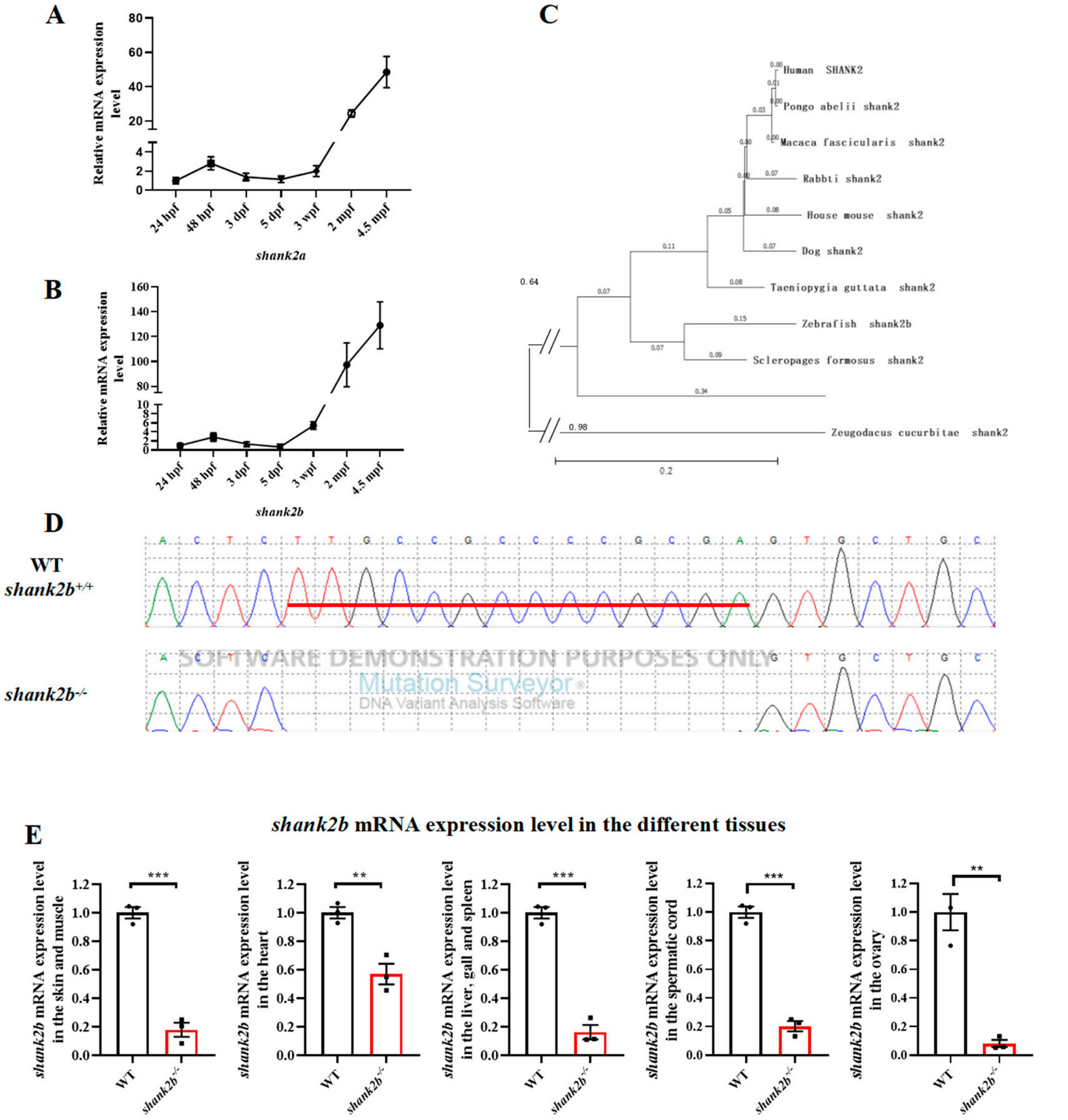

Supplementary Figure S1 *shank2* orthologues are conserved in zebrafish and CRISPR-Cas9 induced *shank2b* target mutation. A-B Temporal mRNA expression profiling of zebrafish *shank2a* and *shank2b* at seven stages: 24 hpf, 48 hpf, 3 dpf, 5 dpf, 3 wpf, 2 mpf and 4.5 mpf. Data are shown as mean ± SEM, n = 3-4. **C** Phylogenetic analysis of SHANK2 in 10 species. The phylogenetic tree was constructed using the neighbor-joining methods as implemented in the MEGA 6 package. All of the sequences are available from the NCBI protein database. *Human*, NP_036441.21, *Pongo abelii*, XP_024111518, *Macaca fascicularis*, XP_015290734.1, *Rabbit*, XP_002723176, *House mouse*, NP_001074839.3, *Dog*, XP_022260831, *Taeniopygia guttata*, XP_030129375, *Scleropages formosus*, XP_029112011.1, *Zeugodacus cucurbitae*, XP_028896935.1, *Danio rerio* shank2a, XP_021323618.1, *Danio rerio* shank2b, NP_001121819.1. The obtained numbers represented evolutionary relationships, with larger numbers indicating greater genetic differences. In vertebrate animals, the genetic distance was not greater than “0.35”. In invertebrates (such as *Zeugodacus cucurbitae*), the distance was as high as “0.98”. **D** Sanger sequence confirmation of 14-nucleotide deletion mutations highlighted in red. **E** Reduced

expression of *shank2b* mRNA in the other tissues of 4.5 mpf male *shank2b*$^{-/-}$ mutants (Skin and muscle, ***p =0.0002; Heart, **p = 0.0065; Liver, Gall and Spleen, ***p = 0.0002). Reduced expression of *shank2b* mRNA in the ovary of female *shank2b*$^{-/-}$ (** p = 0.0021) and spermatic cord of male mutants at 4.5 mpf (***p = 0.0001). Each group n = 3. Student's t test. Data are shown as mean ± SEM.

Supplementary Figure S2

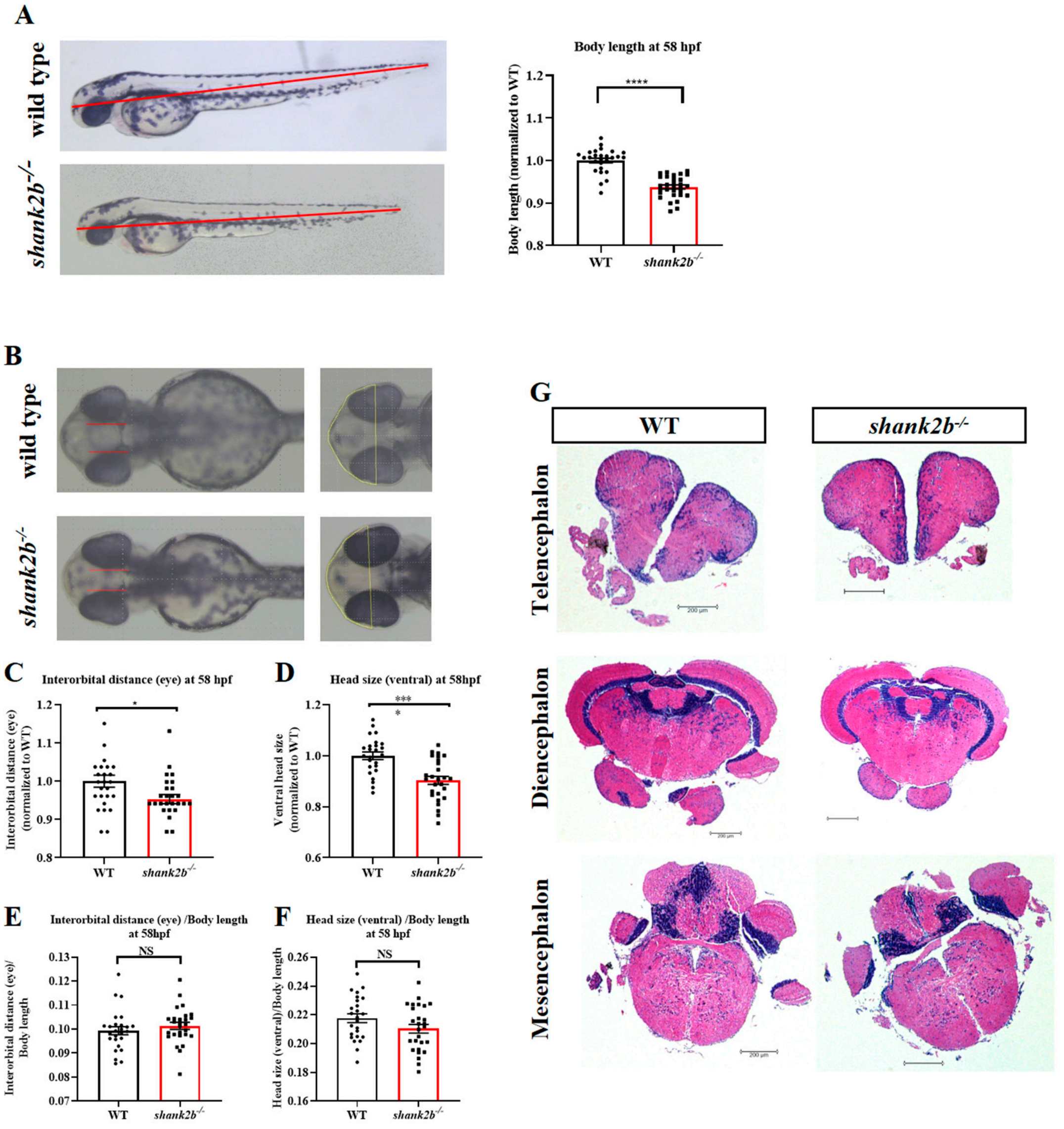

Supplementary Figure S2 Morphological analysis of *shank2b*$^{-/-}$ zebrafish. A The body length of *shank2b* mutants at 58 hpf were shorter in body length than WT larvae (WT n = 27, *shank2b*$^{-/-}$ n = 30, **** $p < 0.0001$, Student's t test). Data are presented as mean ± SEM. **B-F** Head size in wild-type and *shank2b* mutant were measured from dorsal and ventral images using Image J. Representative tracings, the distance between the convex tips of the eyes, done in Image J are shown in red, as well as the boundaries of forebrain-midbrain in yellow. The head size appears to be smaller in mutants **(B, C, D)** larvae at 58 hpf eye interorbital distance, WT n = 26, *shank2b*$^{-/-}$ n = 27, * $p = 0.0222$, Student's t test; ventral head size, WT n = 25, *shank2b*$^{-/-}$ n = 27, **** $p < 0.0001$, Student's t test). **(E, F)** The ratio of Interorbital distance (eye)/ body length (WT n = 26, *shank2b*$^{-/-}$ n = 27, ns, $p = 0.3688$) and Head size (ventral)/ body length (WT n = 25, *shank2b*$^{-/-}$ n = 27, ns, $p = 0.0947$, Student's t test) at 58 hpf between WT and mutants showed no significant difference. Data are presented as mean ± SEM. **G** Examination of brain tissues in WT (left images) and *shank2b*$^{-/-}$ (right images) by H&E staining at 4.5 mpf. Zebrafish brains were sectioned at telencephalic, diencephalic and mesencephalic levels. Scale bar = 200 μm.

Supplementary Figure S3

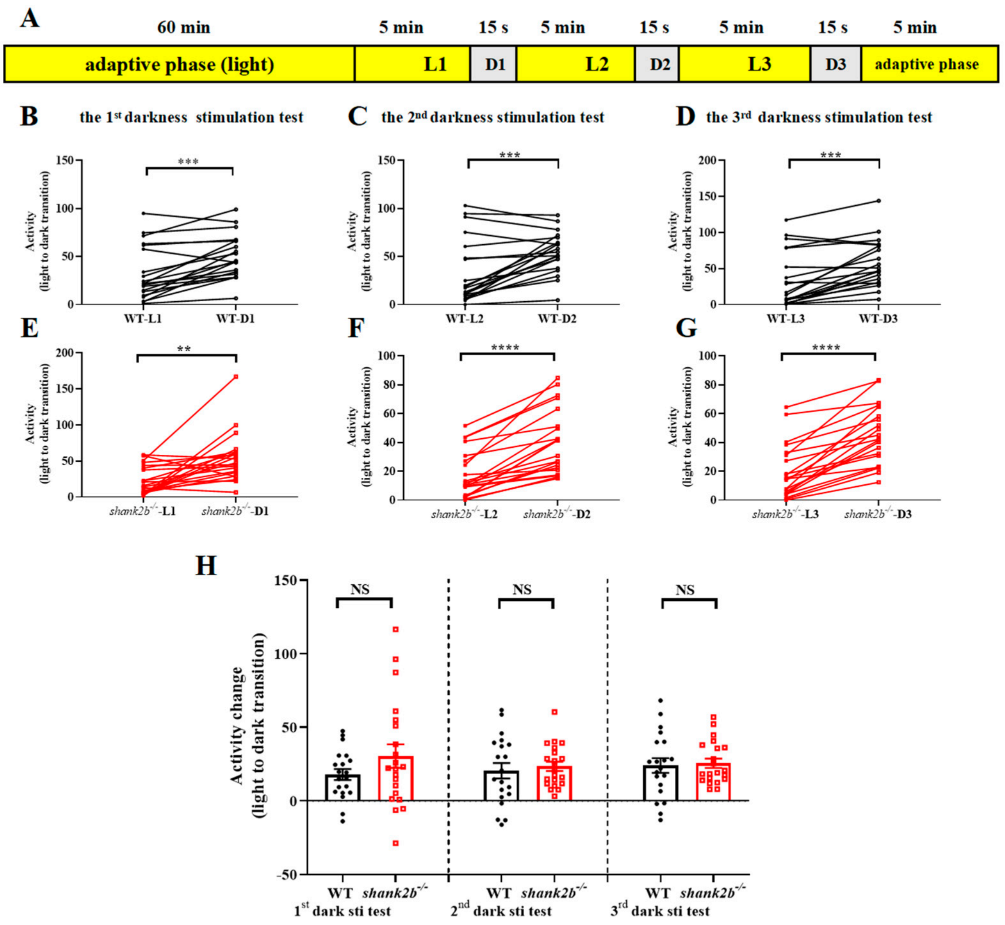

Supplementary Figure S3 *shank2b* mutants exhibited normal VMR response to darkness stimuli. A Scheme and behavioral setup applied for locomotor activity tracking in VMR response to darkness stimulation of zebrafish larvae at 13 dpf. The experiment consisted of one 60-min adaptation period under continuous illumination and one 20-min 45-s testing period consisting of three VMR tests. One VMR experiment consisted of 5 min of conditioning to illumination and 15 s of stimulation by a sudden darkness stimuli. **B-G (E-G)** *shank2b*$^{-/-}$ models exhibited normal response (first dark stimuli, ** p = 0.001; second dark stimuli, **** p <0.0001; third dark stimuli, **** p <0.0001. n = 21, Paired t test), characterized by dramatic increases in movement in response to three sudden transitions from light to darkness, similar to WT larvae **(B-D)** (first dark stimuli, **** p <0.0001; second dark stimuli, *** p =0.0009; third dark stimuli, *** p = 0.001; n = 20, Paired t test). **(H)** Column plots compare activity detected during the 1 min before and the 15 s after each darkness stimulation exposure between WT and *shank2b* mutants. Both WT and *shank2b* larva were sensitive to dark stimuli, manifested as no significant difference in activity change three times from light to dark (first dark stimuli, ns p = 0.1667; second dark stimuli, ns p = 0.6332; third dark stimuli, ns p = 0.7971; WT n = 20, *shank2b*$^{-/-}$ n = 21, Student's t test). Data are presented as the mean ± SEM.

Supplementary Figure S4

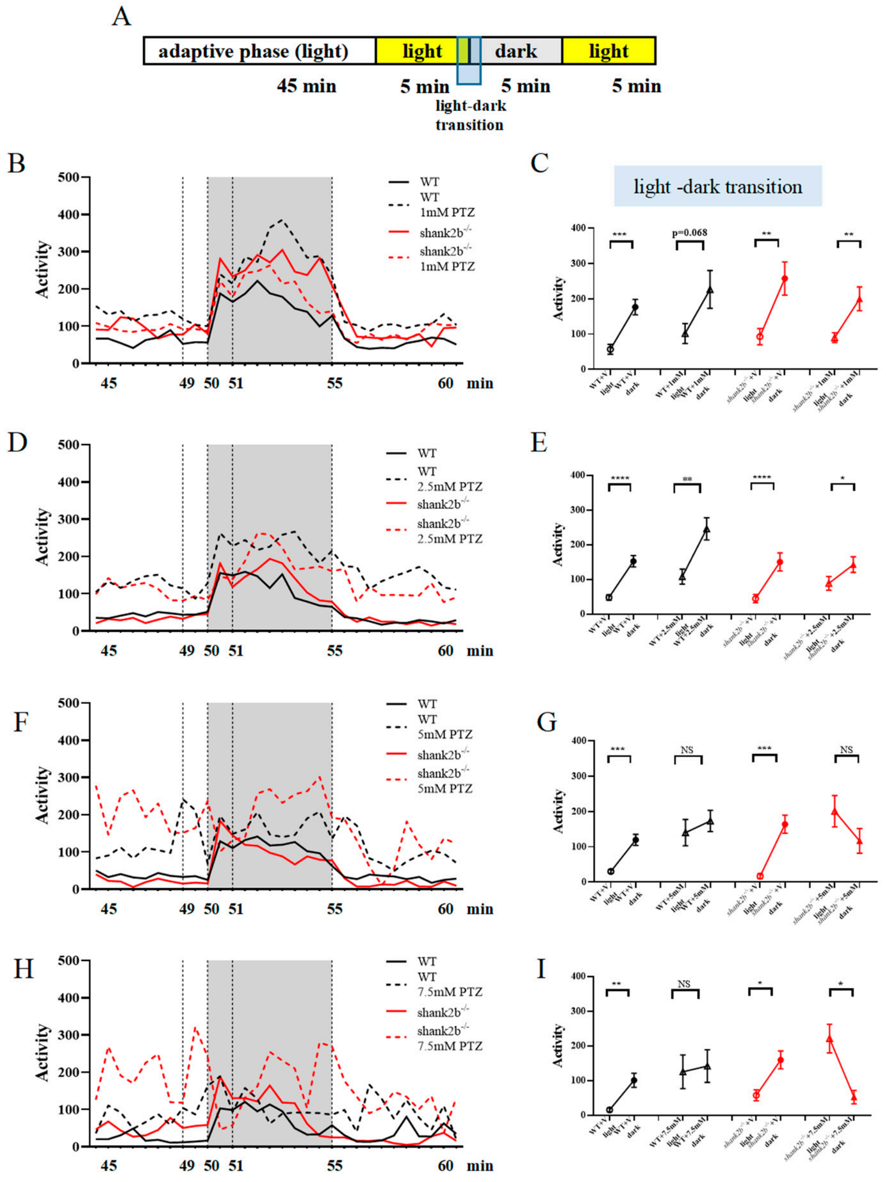

Supplementary Figure S4 Dose-response paired plots showing the change in activity during light to dark transition per fish of wild-type or *shank2b*$^{-/-}$ fish at 9dpf exposed to water or increasing concentrations of pentylenetetrazol (PTZ) for 1 h. A PTZ dose-response behavioral experimental procedure. The experiment consisted of a 45-minadaptation period of light condition and a 15-min testing period consisting of one light-dark cycle, consisted of 5 min of conditioning to light and 5 min of darkness stimulation. **B-I** Experiments were performed at 9 dpf. **(B, D, F, H)** The horizontal axis denotes the experimental progression of the testing period. The vertical axis denotes the activity detected by larvae in each 30-seconds time bin. PTZ concentration information is marked on the top right corner of each panel. Data are presented as the mean. **(C, E, G, I)** Effects of PTZ treatment on locomotor activity during light-dark transitions. Paired dot plots compared average swimming distances per larva of the light conversions in the one min before and after the light-to-dark conversions. Data are presented as the mean ± SEM. **(C)** PTZ concentrations of 1 mM of WT and ***shank2b***$^{-/-}$ fish exhibited an

dynamic increase of activity during light-dark transitions, which means that no change had been induced compared with untreated larvae at 9 dpf (WT n = 12, *** $p = 0.0002$; WT + 1 mM n = 11, ns, $p = 0.0668$; *shank2b*$^{-/-}$ n = 10, ** $p = 0.0052$; *shank2b*$^{-/-}$ + 1 mM n=12, ** $p = 0.0062$; Paired t test). **(E)** PTZ concentrations of 2.5 mM of WT and ***shank2b***$^{-/-}$ fish didn't elicit a decline in activity (WT n = 18, **** $p < 0.0001$; WT + 2.5 mM n = 18, ** $p = 0.0022$; *shank2b*$^{-/-}$ n = 18, **** $p < 0.0001$; *shank2b*$^{-/-}$ + 2.5 mM n=18, * $p = 0.0459$; Paired t test). **(G)** The activity of PTZ-treated WT larvae also increased at concentrations of 5 mM, whereas 5 mM PTZ-treated *shank2b*$^{-/-}$ slightly decreased. Although these results did not achieve statistical significance (WT n = 16, *** $p = 0.0001$; WT + 5 mM n = 15, ns, $p = 0.4365$; *shank2b*$^{-/-}$ n = 12, *** p =0.0001; *shank2b*$^{-/-}$ + 5 mM n=12, ns, $p = 0.1393$; Paired t test). **(I)** 7.5 mM PTZ-treated *shank2b*$^{-/-}$ significantly decreased during the light-dark transition, whereas 7.5 mM PTZ-treated WT larvae also increased without statistical significance (WT n = 6, ** $p = 0.0039$; WT +7. 5 mM n = 6, ns, $p = 0.7292$; *shank2b*$^{-/-}$ n = 6, * p =0.0136; *shank2b*$^{-/-}$ + 7.5 mM n=6, * $p = 0.0292$; Paired t test).

Supplementary Figure S5

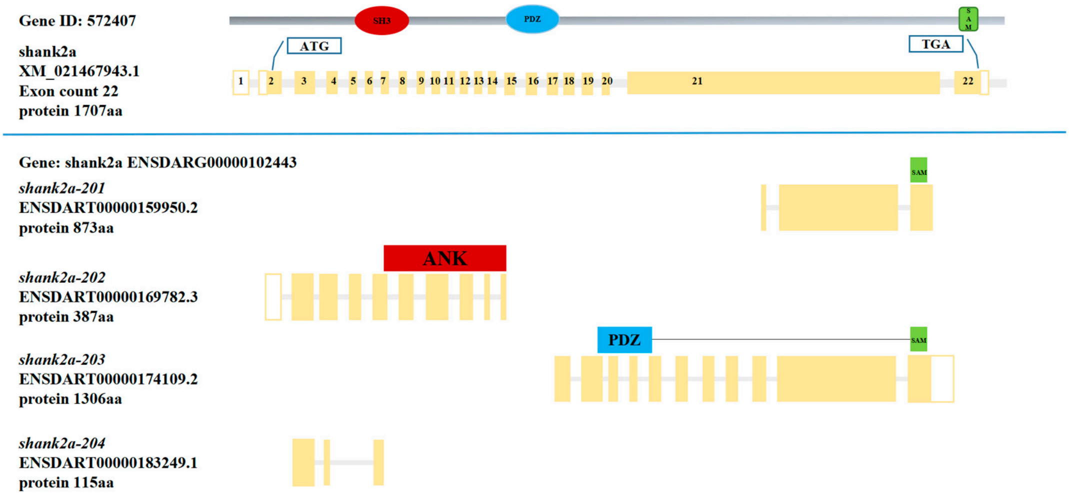

Supplementary Figure S5 Different isoforms of zebrafish *shank2a*. Sequence data from NCBI and Ensembl

Supplementary Figure S6

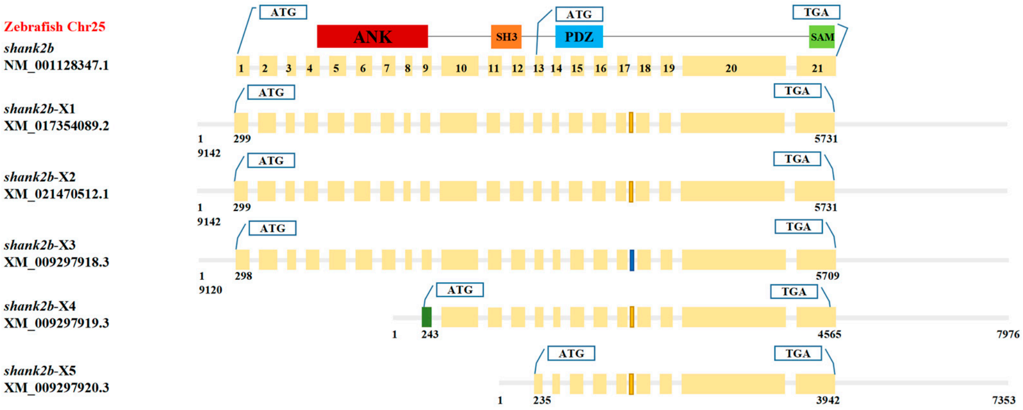

Supplementary Figure S6 Different isoforms of zebrafish *shank2b*. Sequence data from NCBI
